# Seedling responses to salinity of 26 Neotropical tree species

**DOI:** 10.1093/aobpla/plz062

**Published:** 2019-11-25

**Authors:** A De Sedas, Y González, K Winter, O R Lopez

**Affiliations:** 1 Centro de Biodiversidad y Descubrimiento de Drogas, Instituto de Investigaciones Científicas y Servicios de Alta Tecnología, Ciudad del Saber, Clayton, Panamá, República de Panamá; 2 Department of Biotechnology, Acharya Nagarjuna University, Guntur, Andhra Pradesh, India; 3 Smithsonian Tropical Research Institute, Balboa, Ancón, Panamá, República de Panamá

**Keywords:** Mortality, photosynthesis, relative growth rate, salt tolerance, stomatal conductance, tropical trees

## Abstract

Sea-level rise will result in increased salinization of coastal areas. Soil salinity is a major abiotic stress that reduces plant growth, yet tolerance to salinity varies across environmental conditions, habitats and species. To determine salinity tolerance of 26 common tropical tree species from Panama, we measured growth, gas exchange and mortality of 3-month-old seedlings subjected to weekly irrigation treatments using five seawater solutions (0 % = control, 20, 40, 60 and 90 % V/V of seawater) for ~2 months. In general, species from coastal areas were more tolerant to increased seawater concentration than inland species. Coastal species such as *Pithecellobium unguis-cati*, *Mora oleifera*, *Terminalia cattapa* and *Thespesia populnea* maintained growth rates close to those of controls at 90 % seawater. In contrast, inland species such as *Minquartia guainensis*, *Apeiba membranacea*, *Ormosia coccinea* and *Ochroma pyramidale* showed strong reductions in growth rates and high mortality. Plant height and leaf production also differed greatly between the two groups of plants. Furthermore, measurements of gas exchange parameters, i.e. stomatal conductance and maximum photosynthetic rate, were consistent with the contrasting growth responses of coastal and inland species. Our research reveals a great degree of variation in salinity tolerance among tropical tree species and demonstrates a close relationship between species habitat and the ability to thrive under increasing salt concentration in the soil, with coastal species being better adapted to withstand increased soil salinity than non-costal species.

## Teaser

We examined salinity tolerance among 26 common tropical tree species found along coastal and inland wet forests of Panama. While salinity tolerance varies greatly among tropical tree species we found a close relationship between species habitat and the ability to thrive under increasing salt concentration in the soil, with coastal species being better adapted to withstand increased soil salinity than non-coastal species.

## Introduction

Climate change is causing sea level to rise (IPCC 2012; Copping *et al.* 2018). Increased flooding in coastal areas will add to soil salinization, which currently affects >30 % of the world’s arable land (FAO 2011). Salt intrusion, as a consequence of sea-level rise, is already causing shifts in agricultural practices in the Mekong river delta, one of the largest rice-producing regions in the world (Renaud *et al.* 2013). Hence, increased soil salinity due to climate change will exacerbate earth’s sustainability, undermining food security as soil salinity reduces crop yield (Wheeler and von Braun 2013). At a broader scale, the resilience and productivity of adjacent coastal plant communities will also be compromised as sea-water flooding alters the overall biogeochemistry of soils, limiting carbon sequestration, and affecting greenhouse gases emissions and other ecosystem services (Patel and Pandey 2007; Godfray *et al.* 2010).

Salinity represents a major problem for many plants, e.g. non-halophytes (Setia *et al.* 2012; Zandalinas *et al.* 2018). As the concentration of soluble salts in the soil increases, plant functioning is compromised via osmotic imbalance, increased production of reactive oxygen species (ROS, oxidative stress) and salt ion toxicity (Gill and Tuteja 2010). In the absence of osmotic adjustments and compartmentalization of solutes by plants, increased salinity may lead to reduced carbon assimilation via stomatal closure (Shekoofa *et al.* 2013). As ion accumulation increases, the generation of ROS disrupts cellular processes leading to leaf chlorosis and lastly ion toxicity causing plant death (Walker *et al.* 1979). While salinity tolerance requires a concatenation of complex physiological processes, quantifying salinity tolerance among plants has traditionally relied on assessing growth under increased salt ions in the soil (Ordoñez *et al.* 2018).

In addition to climate change, salinization is directly associated to human-derived activities such as deforestation, over irrigation for nutrient-enriched soils and inefficient urban planning (Oldeman 1992; Nicholls and Lowe 2004). Restoration of salty soils can be achieved through phytoremediation, i.e. the use of woody or non-woody salt-tolerant plant species that can sequester salt ions from saline soils (Munns and Tester 2008; Rich *et al.* 2017). Limited information is available on tropical woody trees’ capacity to deal with increased salt ion in the soil. Most studies on salinity tolerance involved tree species of known economic importance such as *Citrus* spp. (Storey and Walker 1998), avocado (*Persea americana*) (Mickelbart and Arpaia 2002), eucalyptus (*Eucalyptus grandis*) (Sun and Dickinson 1995), olive tree (*Olea europaea*), pine (*Pinus radiata*) (Sala 2009) and pistachio (*Pistacia vera*) (Rahneshan *et al.* 2018), among others. Less is known about the ecology of the species, species traits or mutualistic interactions in their response to salinity. For example, salinity tolerance of *Malus domestica* (apple tree), an exclusive crop of temperate zones, is achieved by combining biochemical control of osmoregulation and reactive oxygen species (ROS), and physiological and structural adaptations are widely recognized in alleviating soil salt stress when the proper mycorrhizal lines are used (Kapoor *et al.* 2013). This research aims to fill this gap by examining the salt tolerance of 26 tree species that occur in the neotropics.

Coastal marine conditions influence tree species distribution (Bijlsma *et al.* 1995). Coastal tree species can also occur in inland forests, while most inland species typically do not occur in or near marine-influenced habitats. This led us to hypothesize that coastal tropical tree species are relatively salt-tolerant while most inland forest species are intolerant to salinization. Our research has two main objectives: (i) determine growth responses to salinity of a range of common tropical tree species from coastal and inland areas, and (ii) use this information to generate a tropical tree species versus salinity tolerance ranking as a resource for mitigation strategies in the face of future sea-level rise scenarios.

## Materials and Methods

### Study site

Experiments were conducted in a greenhouse facility located 25 km north of Panama City, in Gamboa (09°07′N, 79°42′W), operated and maintained by the Smithsonian Tropical Research Institute. Plants were cultivated underneath a glass-roof that prevented rainfall from entering the pots. Plants grew under close to full sunlight conditions, natural air circulation, and natural temperature and relative humidity conditions.

### Species selection and plant material

Twenty-six species of trees native or naturalized to Panama, from coastal and inland forests, were chosen depending on seed availability. Species represent a diversity of families and distribution ranges (Table 1). Seeds were collected from at least three mature individuals occurring in the same area. We placed seeds in germination trays in a shaded greenhouse at 35 % of natural sunlight. After germination, seedlings with two or more fully expanded and mature leaves were transferred to 2.3-L pots (Stuewe and Sons, Corvallis, OR, USA), filled with forest soil. Seedlings were 3 months old when subjected to experimentation.

**Table 1.** Coastal and inland tree species used in this study including family, location of seed material, forest type and geographical distribution. Species authorities, family and name nomenclature follows (Correa *et al.* 2004). *Non-native naturalized species. Forest types: WF (wet forest), DF (dry forest), CF (cloud forest), AS (Atlantic slope), PS (Pacific slope).

Species	Family	Coordinates	Forest type	Plant distribution
COASTAL SPECIES				
*Terminalia catappa*	Combretaceae	8°59′2″N, 79°32′0″W	CF AS PS	Tropics
*Mora oleifera*	Fab. Caesalpinioideae	7°56′24″N, 81°17′54″W	CF AS PS	Costa Rica to Colombia
*Pithecellobium unguis-cati*	Fab. Mimosoideae	8°53′35″N, 79°39′20″W	CF PS	Mexico to Venezuela and Caribbean
*Thespesia populnea*	Malvaceae	8°54′28″N, 79°31′32″W	DF WF AS PS	Tropics
*Sterculia apetala*	Malvaceae	8°53′35″N, 79°39′20″W	DF WF AS PS	Mexico to Brazil
*Cedrela odorata*	Meliaceae	8°59′2″N, 79°32′0″W	DF WF AS PS	Mexico to Argentina
INLAND SPECIES				
*Anacardium excelsum*	Anacardiaceae	9°04′24″N, 79°39′57″W	DF WF AS PS	Honduras to Ecuador
*Annona muricata*	Annonaceae	8°45′0″N, 79°54′0″W	WF AS PS	Tropical America*
*Cananga odorata*	Annonaceae	8°59′2″N, 79°32′0″W	WF AS PS	Old tropics*
*Tabebuia rosea*	Bignoniaceae	9°7′0″N, 79°42′0″W	DF WF AS PS	Mexico to Ecuador
*Protium pecuniosum*	Burseraceae	9°19′28″N, 82°32′27″W	WF AS	Costa Rica to Panama
*Adenanthera pavonina*	Fab. Mimosoideae	9°7′0″N, 79°42′0″W	WF AS PS	Tropical America
*Calliandra trinervia*	Fab. Mimosoideae	9°7′0″N, 79°42′0″W	WF AS PS	Tropical South America*
*Enterolobium cyclocarpum*	Fab. Mimosoideae	8°00′38″N, 80°29′08″W	DF WF AS PS	Mexico to Brazil
*Erythrina costaricensis*	Fab. Papilionoideae	9°7′0″N, 79°42′0″W	DF WF AS PS	Costa Rica to Colombia
*Inga* sp.	Fabaceae/mim.	9°19′28″N, 82°32′27″W	WF AS	Panama
*Ormosia coccinea*	Fabaceae/pap.	9°7′28″N, 79°42′55″W	WF AS	Nicaragua to Brazil
*Guazuma ulmifolia*	Malvaceae	9°7′0″N, 79°42′0″W	DF WF AS PS	Mexico to Argentina
*Luehea seemannii*	Malvaceae	9°7′0″N, 79°42′0″W	DF WF AS PS	Belice to Venezuela
*Ochroma pyramidale*	Malvaceae	9°7′0″N, 79°42′0″W	DF WF AS PS	Mexico to Bolivia and Caribbean
*Apeiba membranacea*	Malvaceae	9°19′28″N, 82°32′27″W	WF AS	Honduras to Brazil
*Swietenia macrophylla*	Meliaceae	8°59′2″N, 79°32′0″W	DF WF AS PS	Mexico to Bolivia
*Castilla elastica*	Moraceae	9°7′28″N, 79°42′55″W	WF AS PS	Nicaragua to Brazil
*Virola koschnyi*	Myristicaceae	9°19′28″N, 82°32′27″W	WF AS	Guatemala to Panama
*Minquartia guianensis*	Olacaceae	9°19′28″N, 82°32′27″W	WF AS	Nicaragua to Brazil
*Faramea eurycarpa*	Rubiaceae	9°7′28″N, 79°42′55″W	WF AS PS	Costa Rica to Ecuador

### Experimental design and salt treatments

We employed a randomized design with five seawater concentrations. Plants were treated with 150 mL of 20, 40, 60 and 90 % of seawater, while control plants (0 % seawater) were irrigated with 150 mL of tap water, respectively, once a week. We used 8–10 replicates for each treatment (total of 40–50 plants per species). We ran experiments in consecutive phases depending on seedling availability. In addition to flushing soils with treatment solutions, plants received 50 mL of tap water every 2 days to replace water lost by evaporation and transpiration.

### Growth measurements

For all 26 species we measured plant height and leaf number every 15 days. Plant height was measured from the base of the stem to the tallest bud using a metered ruler. The total number of leaves was counted during each census. In the case of species with compound leaves, leaflets were counted as leaves. We measured initial and final biomass as the sum of dry mass of leaves, stems (including petioles of the leaves) and roots after drying for 72 h at 70 °C. Total leaf area was determined using a LiCor 3000 area meter (LiCor Instruments, Lincoln, NE, USA). Initial and final seedling total biomass was used to determine the relative growth rate (RGR), defined as:

$$\begin{array}{l} RGR(g\;{g^{-}}^{1}\;da{y^{-}}^{1})=(lnW_{2}-lnW_{1})/t, \end{array}$$

where *W*_1_ is the initial total dry mass in g and *W*_2_ is the total dry mass at final harvest in g, and *t* is the time of the treatment in days.

### Survivorship

The number of plants that was still alive at the end of experiments, 60 ± 2 days, was used to determine plant survivorship across all species. A plant was considered dead after all leaves had been dropped and the stem was dry.

### Stomatal conductance and maximum photosynthetic rate

Stomatal conductance (*g*_s_) and maximum photosynthetic rates (*A*_max_) were measured in a subset of coastal and inland species with leaves that allowed for easy insertion into the LI-6400 photosynthesis chamber (6 cm^2^). Coastal species included: *Mora oleifera Thespesia polpunea and Cedrela odorata*, while inland species included *Guazuma ulmifolia*, *Faramea eurycarpa*, *Cananga odorata*, *Protium pecuniosum*, *Luehea seemannii*, *Ochroma pyramidale*, *Castilla elastica*, *Apeiba membranacea* and *Virola koschnyi*. Measurements of leaf stomatal conductance (*g*_s_) and maximum photosynthetic rates (*A*_max_) were made using a LiCor 6400 portable photosynthesis system (LI-6400, LiCor Instruments, Lincoln, NE, USA). Both *g*_s_ and *A*_max_ were obtained by illuminating a healthy, fully expanded leaf at 1000 μmol m^−2^ s^−1^ of photosynthetic photon flux density (PPFD), until a steady state of net CO_2_ fixation and stomatal conductance was reached. Artificial illumination was supplied to the leaf from the LI-6400’s red-blue LED light source, chamber temperature was kept at 28 °C, a CO_2_ reference partial pressure of 400 μmol·mole of CO_2_ was maintained by the LI-6400 CO_2_ mixer and leaf chamber relative humidity was maintained above 80 %.

### Statistical analysis

Plant height, leaf number, RGR, survival percentage, *g*_s_ and *A*_max_ were used as response variables by species across seawater treatments. We applied an analysis of variance (ANOVA) to detect seawater concentration treatment effects among the studied species using each of the response variables independently. An additional ANOVA was done to assess species and habitat effects, possible interaction of the two and their effects on RGR and mortality. For assessing treatment effects on gas exchange parameters *g*_s_ and *A*_max_ we employed a repeated measured ANOVA. A hierarchical cluster analysis was performed as an exploratory tool in order to visually rank salinity tolerance among all studied species regardless of the habitat they came from (coastal and non-coastal) and summarize species response to salinity in a concise form using all parameters, except *g*_s_ and *A*_max_. This analysis is represented in a cladogram that shows how the 26 species ranked using their response to 90 % seawater. All ANOVA analyses were conducted using JMP v 13 (SAS Institute, Cary, NC, USA). The hierarchical cluster and cladogram analyses were done using IBM SPSS Statistics version 22 (IBM Corporation, Armonk, NY, USA). All graphs of growth and physiological responses depicting averages and standard errors were done using Sigma Plot 12.3 (Systat Software, Inc., San Jose, CA, USA).

## Results

### Plant height, leaf number and RGR

**Figure 1.**
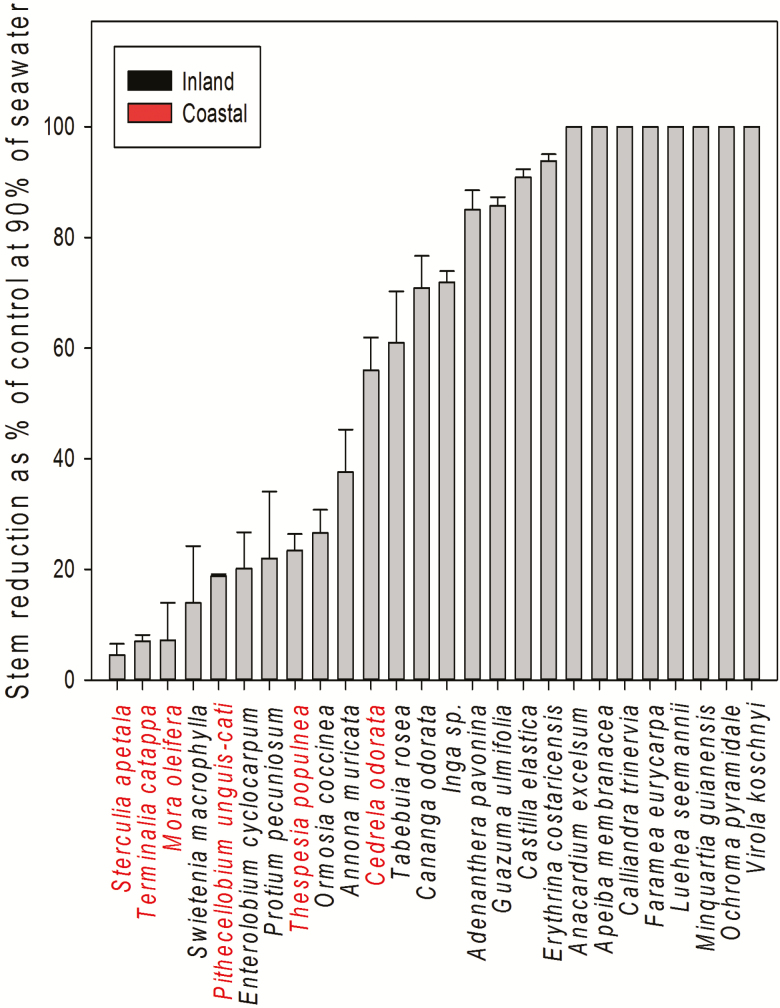
Stem height reduction at 90 % of seawater irrigation treatment (±SE) in coastal and inland species. See **Supporting Information** for all other seawater treatments figures.

Across all species, plant height was significantly reduced by seawater treatment starting at 30 days (*F*_4, 25_ = 14.8, *P* < 0.0001 ANOVA). For example, on average, regardless of species, seedlings subjected to 90 % of seawater irrigation were 40 % shorter than those under control (LSMeans Tukey HSD, Fig. 1, **see****Supporting Information—Fig. S1**, for all other seawater treatments). However, reduction of plant height in response to seawater treatment varied among species (*F*_4, 25_ = 78.1, *P* < 0.0001). Just after 30 days of irrigation with 20 % seawater, plant height was significantly reduced in the inland species. *Castilla* was the most sensitive species among all, showing a stem height reduction near 75 % after 4 weeks of being irrigated with 20 % seawater (LSMeans Tukey HSD). Similarly, seedlings of *Apeiba*, a species common to inland mature tropical wet forests, showed 30 % stem height reductions after 30 days under the same seawater treatment. In contrast, coastal species such as *Thespesia*, *Terminalia*, *Mora* and *Pithecellobium* showed no reduction in stem height when compared to the control at the end of the experiment under 90 % concentration of seawater. In fact, seedlings of *Pithecellobium* under 20 % seawater grew 4 % more than control seedlings.

**Figure 2.**
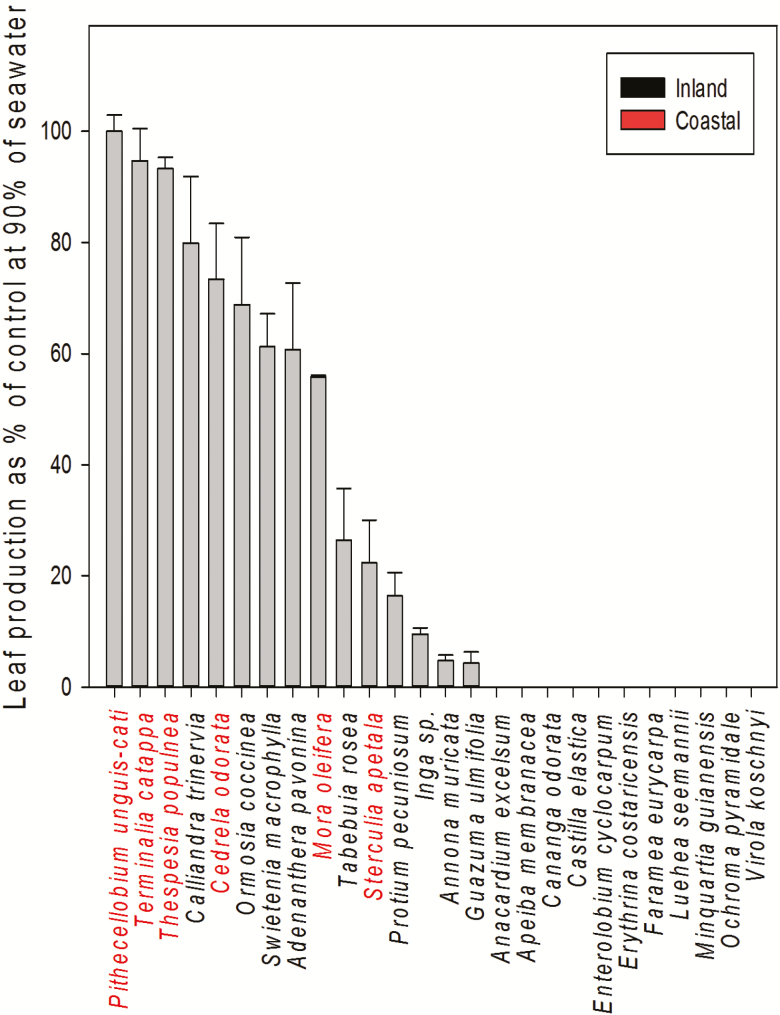
Leaf production at 90 % of seawater irrigation treatment (±SE) in coastal and inland species.

Leaf number also decreased significantly as seawater concentration increased after 30 days across all species (*F*_4, 25_ = 8.47, *P* < 0.0001, Fig. 2). However, increased seawater solution affected species differently (*F*_4, 25_ = 31.8, *P* < 0.0001). Among inland species, seedlings of *Apeiba* showed a 47 % reduction in leaf number under 20 % of seawater, but all the leaves had been dropped after 4 weeks at 40, 60 and 90 % seawater treatments. Coastal species *Thespesia*, *Terminalia* and *Sterculia* showed no significant reduction in leaf number after 60 days across seawater treatments, including 90 % (LSMeans Tukey HSD). However, *Mora* and *Cedrela* showed reduction in leaf number after 60 days of exposure to 60 % seawater treatment.

**Figure 3.**
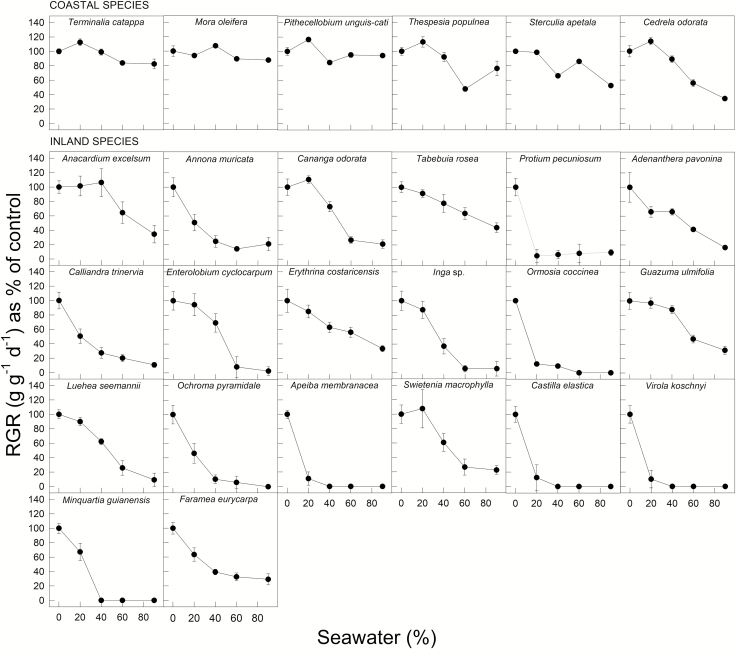
Relative growth rates as percentage of control (±SE) for all studied species across seawater treatments. Panels are arranged in relation to Table 1.

Relative growth rate was much less affected by salinity in the coastal species than in the inland species (Fig. 3, for absolute RGR values, **see****Supporting Information—Fig. S2**). Inland species had significantly lower RGRs than coastal species regardless of seawater treatments (*F*_1, 4_ = 159.5, *P* < 0.0001). When compared to controls, seedlings of *Virola*, *Apeiba* and *Castilla* grew just 15 % under 20 % seawater, and above this treatment all plants died. In contrast, seedlings of *Mora*, *Thespesia*, *Terminalia* and *Pithecellobium* maintained above 75 % of RGR under 90 % of seawater when compared to controls. However, *Cedrela*, a dry forest species, showed significant reductions in RGR at 90 % of seawater **[see****Supporting Information—Fig. S3****]**.

### Survival under salinity

**Figure 4.**
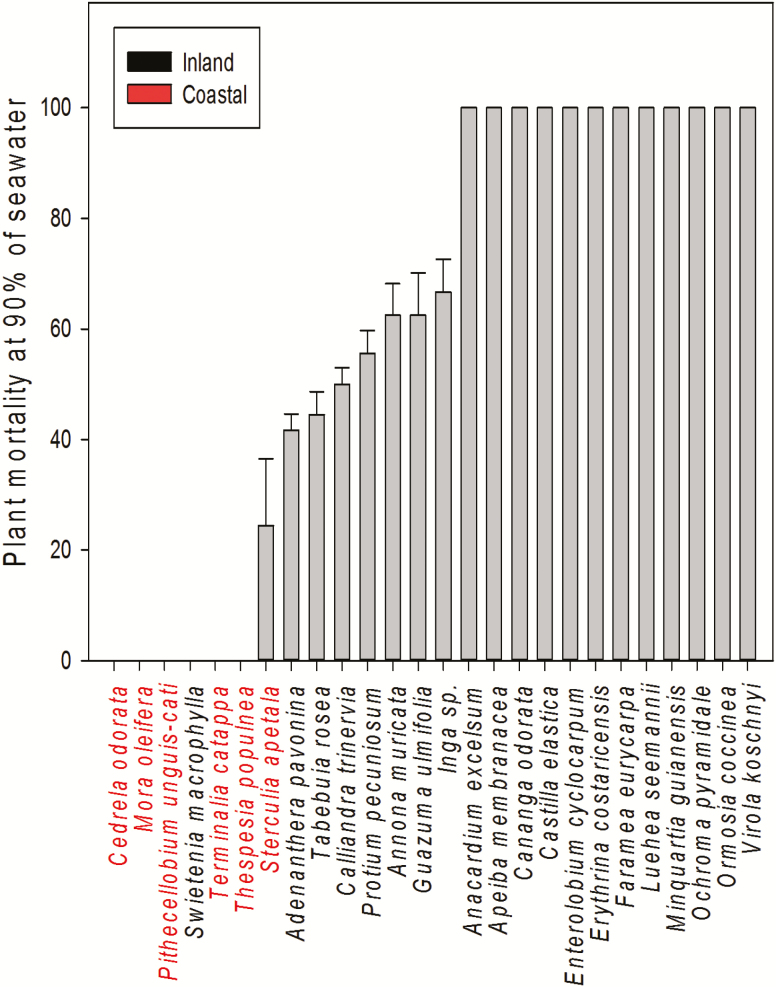
Plant mortality by species given as percentage under 90 % seawater irrigation treatment (±SE) for coastal and inland species. Panel letters indicate groupings according to their tolerance.

Seedling mortality for coastal and inland species increased as treatment increased, particularly when concentrations surpassed 40 % of seawater (*F*_1, 4_ = 159.5, *P* < 0.0001). However, seedling mortality depended on the interaction of species habitat and treatment (*F*_1, 4_ = 5.1, *P* < 0.001). The coastal species *Thespesia*, *Terminalia*, *Pithecellobium*, *Mora* and *Cedrela* had 100 % plant survival, even at 90 % seawater concentration. On the contrary, seedlings of *Apeiba*, *Virola*, *Castilla*, *Minquartia* and *Ochroma* species showed about 100 % mortality at 90 % seawater concentrations, respectively (LSMeans Tukey HSD). Among the inland species, *Guazuma*, *Annona* and *Inga* showed between 33 and 38 % survival at 90 % sweater concentration (Fig. 4).

### Stomatal conductance and maximum photosynthetic rate

**Figure 5.**
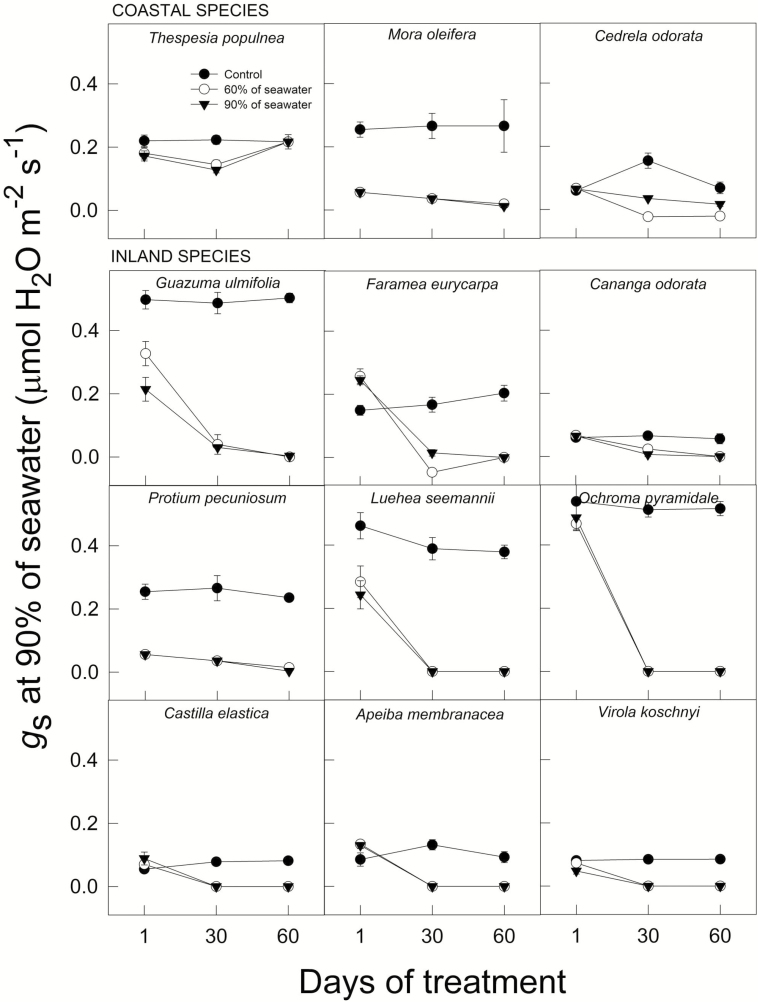
Stomatal conductance (*g*_s_) across time (±SE) under control conditions, and at 60 and 90 % of seawater for coastal and inland species. Data represent averages of 3–5 individuals.

Across all species, increased salinity significantly reduced stomatal conductance (g_s_) (*F*_4, 247_ = 55.95, *P* < 0.0001, MANOVA). However, salinity affected species differently (species * treatment interaction, *F*_4, 247_ = 10.94, *P* < 0.0001). Both, inland and coastal species, showed significantly lower *g*_s_ after 30 days, as seawater concentration exceeded 20 % (*F*_4, 225_ = 18.74 and *F*_4, 72_ = 27.23, *P* < 0.0001 for inland and coastal species, respectively, one-way ANOVA). Inland species such as *Lueh*a, *Apeiba*, *Ochroma* and *Virola* suffered up to 95 % reductions in *g*_s_, after 30 days starting at 40 % of seawater (Fig. 5). In contrast, *Thespesia*, a species common to coastal areas, did not show reduction in *g*_s_ in any treatment, although *g*_s_ was lowered in seedlings of *Mora* and *Cedrela*, after 30 days when exposed to 90 % of seawater treatment, but such differences were not significant (Fig. 5).

**Figure 6.**
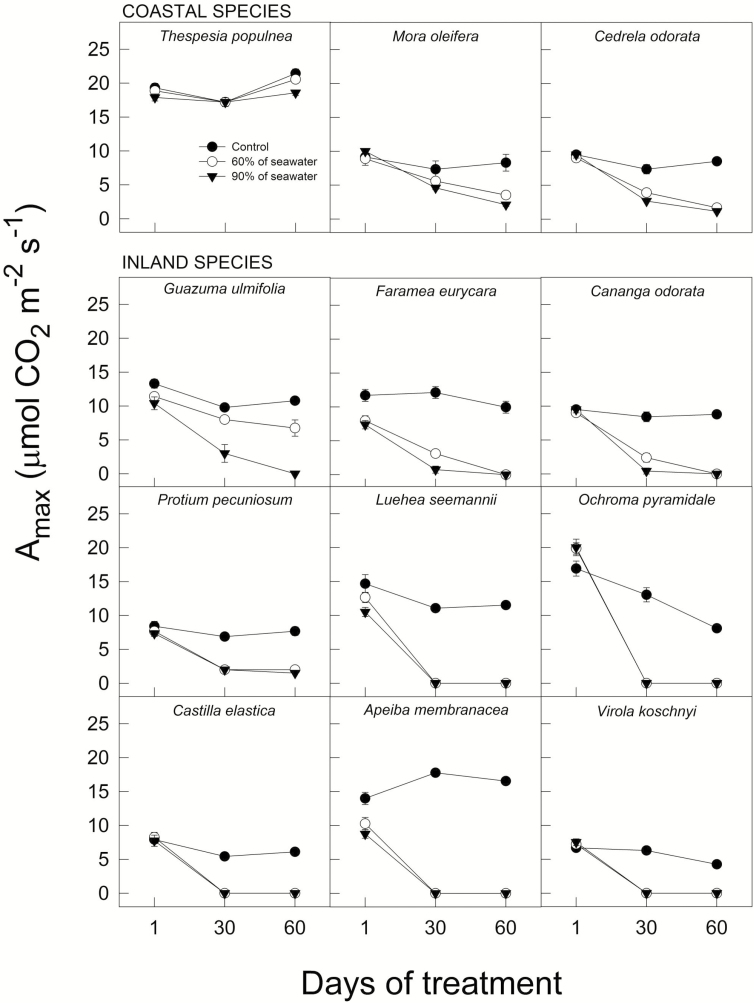
Maximum photosynthetic rate (*A*_max_) across time (±SE) under control conditions, and at 60 and 90 % of seawater for coastal and inland species. Data represent averages of 3–5 individuals.

Maximum photosynthetic rates (*A*_max_) followed a similar pattern than *g*_s_, decreasing as salinity increased (*F*_4, 247_ = 93.08, *P* < 0.0001, MANOVA), with species responding differently to salinity (species * treatment interaction, *F*_4, 247_ = 18.58, *P* < 0.0001). Although *A*_max_ of inland and coastal species was significantly lowered after 30 days (*F*_4, 225_ = 50.54, *P* < 0.0001 and *F*_4, 72_ = 4.55, *P* < 0.005, for inland and coastal species, respectively, one-way ANOVA), the sweater concentration at which *A*_max_ was lowered differed between the two habitats. After 30 days, inland species showed significantly lower *A*_max_ when seawater concentration exceeded 20 % while for the coastal species treatment effects were detectable after 40 % seawater. *A*_max_ varied greatly across species with seedlings of inland species such as *Apeiba*, *Virola*, *Castilla* and *Ochroma*, having >95 % reductions after 30 and 60 days, regardless of treatment (Fig. 6). In contrast, seedlings of the coastal species *Thespesia* showed no remarkable reduction in *A*_max_ across seawater concentration (Fig. 6).

### Species ranking and tolerance thresholds

**Figure 7.**
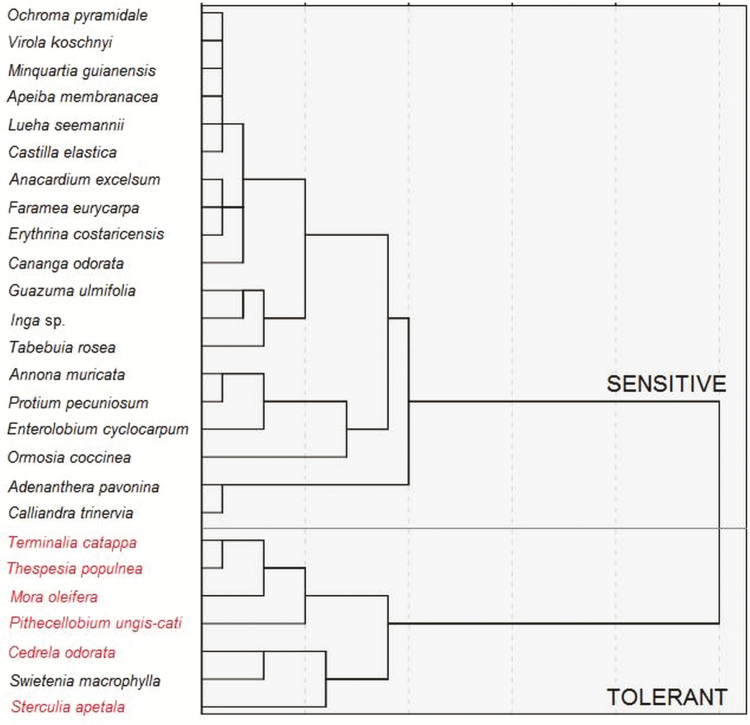
Cladogram representing species salinity tolerance ranking according to a hierarchical clustering analysis using all response parameters, except *g*_s_ and *A*_max_, under 90 % seawater treatment. Within each clade, species are arranged by ascending ranking of salinity tolerance.

To transform multidimensional salinity responses into a reliable ranking of species tolerance, we employed a hierarchical analysis (HA) methodology using all response parameters under 90 % of seawater concentration, with the exception of *g*_s_ and *A*_max_. We excluded the gas exchange parameters as a number of species were not measured and HA is particularly sensitive to missing data. This analysis corroborates the existence of two main clusters that grouped species salinity response into; sensitive and tolerant (Fig. 7, **see****Supporting Information—Fig. S4** for the resulting analysis using gas exchange parameters). The sensitive cluster appears as a relatively heterogeneous group with inland species, some less tolerant than others: *Ochroma*, *Virola*, *Apeiba*, *Minquartia*, *Luehea*, and *Castilla*, *Guazuma*, *Inga*, *Tabebuia*, *Annona*, *Protium*, *Enterolobium*, *Ormosia*, *Adenanthera* and *Calliandra*. And finally, a tolerant cluster readily identified by mostly coastal species such as; *Terminalia*, *Thespesia* and *Pithecellobium* and a two dry forests species, *Cedrela* and *Swietenia*, that maintained RGR under salinity.

## Discussion

This study explores salinity responses among common tropical tree species from coastal and inland habitats in Panama. In general, plant growth decreased as percentage seawater increased across all species, but coastal species were much less affected than inland species. Nearly 20 % of the 26 species evaluated, survived high levels of salinity (Fig. 4). When compared to controls, seedlings of *Mora*, *Pithecellobium*, *Terminalia*, *Thespesia*, *Sterculia* and *Cedrela* maintained above 65 % RGRs when exposed to high salinity irrigation (~30 ‰) (Figs 3 and 4). Among these, *Mora* is a frequent resident of the upper limit of tidal mangroves (Jiménez 1994), which might predispose this species to deal with salinity. Similarly, *Pithecellobium*, *Terminalia* and *Thespesia* are commonly found as marginal vegetation in coastal sandy dunes and beaches (Correa *et al.* 2004), likely experiencing periodic salinity events either by seawater flooding or aerosol deposition from marine spray. Among the coastal species, *Sterculia*, *Swietenia* and *Cedrela* are in fact dry forest species. In the Central American region, dry forests typically occur alongside coastal plains on the pacific slope (Kalacska *et al.* 2004). By contrast, inland species showed severe leaf loss and stagnant plant height that lead to significantly poor performance in RGR **[see****Supporting Information—Appendix S1****]**. For example, *Ochroma* and *Luehea*, common species from gap clearings in tropical wet forests (Croat 1978), showed consistent reduction in RGR in relation to seawater concentration that clearly matches their physiological characteristic. Therefore, the inherently greater growth of coastal species under salinity might be associated to their ecogeographical association to coastal environments, while drought adaptations, i.e. osmotic adjustment, might predispose dry forests species to better deal with salinity conditions. Thus, despite the overall restrictive impact that salinity has on growth, habitat association clearly influences species responses to salinity among tropical tree species.

Reduced stomatal conductance and photosynthesis means limited carbon budgets for plant growth (Duarte *et al.* 2013). We found that RGR varies positively as a function of *A*_max_ across all species and treatments. However, the relationship was only significant after a month under the 20 % seawater treatment, where *A*_max_ explains 30 % of the variance in RGR (*r*^2^ = 0.29, *P* = 0.049). In plants, salinity stress is evidenced by stomatal closure (Lambers 1998). This condition primarily restricts photosynthetic rate (Downton *et al.* 1985) as stomatal closure generates a cascade effect over other physiological processes that impact carbon assimilation. Our results show that coastal species such as *Thespesia* can maintain high rates of photosynthesis and stomatal conductance even at 90 % seawater (Figs 5 and 7). In contrast, inland species such as *Virola*, *Ochroma* and *Apeiba* were among the most salt-sensitive, showing significant reductions in *A*_max_ at high salinity accompanied by low *g*_s_ values (Figs 5 and 7). Previous findings suggest that reductions in CO_2_ assimilation under salinity appear to be initially linked to stomatal closure and not to damage to the photosynthetic machinery (Flexas *et al.* 2004). Some evidence supports the notion that stomatal closure under salinity occurs primarily through the involvement of local synthesis of abscisic acid (ABA), and not necessarily via hydraulic limitations (i.e. turgor loss; Munns and Tester 2008). It is plausible that the first initial response to salt stress observed would be mainly stomatal, resulting in a reduction of the intercellular concentration of CO_2_ (C_*i*_) during photosynthetic CO_2_ uptake, whereas in the long term, as in this study, *g*_s_ and *A*_max_ decrease more or less simultaneously. However, stomatal limitations due to increased ABA or hydraulic limitations via ionic unbalances deserve further attention.

Coastal species under 90 % of seawater salinity showed high survival, for example: *Thespesia*, *Terminalia*, *Pithecellobium*, *Mora* and *Cedrela* which had 100 % of survival (Fig. 4). In contrast, seedlings of inland species *Apeiba* and *Virola*, and many other common to wet forests, had the lowest survival across all seawater treatments, denoting that wet forest species might be inherently sensitive to soil salinity. Plants deal with soil salinity, either by excluding it at its roots or by managing high ionic concentrations. Studies conducted in parallel, using some of the inland species employed here, revealed that many of these highly salt-sensitive tree species are not salt excluders, as high foliar concentrations of Na^+^ and Cl^−^ ions have been measured (A. De Sedas *et al.*, unpubl. data). However, it appears that species associated with dry forests habitats are also capable of enduring salinity conditions. This is the case of *Sterculia*, a species typically found in dry forests surrounding coastal habitats, suggesting a relationship between the proximity such as species occupy from the coastline, drought tolerance and salinity tolerance, as it is expected that coastal and nearby dry forests experience salinity and drought conditions regularly. However, the link between drought and salinity tolerance among tropical tree species remains to be explored.

A major objective of research on plant salinity tolerance has been to improve yield of crop species exposed to salinity stress (Zhu 2001). Little attention has been paid to species-specific life history traits and their relevance under stress, particularly salinity. In this study, *Enterolobium*, a nitrogen (N_2_)-fixing legume from inland dry forests (El-Din *et al.* 2017), maintained relatively good growth up to 40 % seawater, beyond which growth strongly decreased, possibly indicating adverse effects of high soil salinity on the N_2_-fixing bacteria (*Rhizobium*). The importance of effective N_2_-fixing bacteria associations among plant species has been highlighted by Hanin *et al.* (2016), suggesting that salt-tolerant *Rhizobium* strains might significantly help plants cope with increased soil salinity. In the case of tropical trees, it remains to be investigated how salinity responses vary among N_2_-fixing legumes, as no clear pattern emerged from other N_2_-fixing inland species in this study. *Erytrina* appeared to be slightly more salt-sensitive than *Enterolobium*, but *Calliandra* was more tolerant than both (Fig. 7).

The HA supports the idea that coastal species have an advantage over inland species in coping with increased soil salinity (Fig. 7). While our study might be limited from not including species from a broader spectrum of life histories, can salinity tolerance among the study species be explained by species-specific trade-offs? (*sensu*Grime and Pierce 2012). Our HA analysis established clear differences among the study species. The sensitive group is primarily composed, with the exception of *Virola* and *Minquartia*, by fast-growing species characteristic of gap regeneration in tropical forests including: *Ochroma*, *Apeiba*, *Luehea* and *Castilla* (Dalling *et al.* 1998). Such gap-dependent species have been noticeable identified as being highly vulnerable to stressors, for example drought (Engelbrecht *et al.* 2007). By contrast, the most salt-tolerant species within the cladogram are composed of species that inhabit coastal and drought-prone habitats (*Pithecellobium*, *Thespesia*, *Terminalia*, *Mora* and *Cedrela*, *Sterculia* and *Swietenia*, respectively). Further research on tropical tree salinity tolerance based the concept of adaptive strategy theory (Grime and Pierce 2012) may be rewarding.

## Study Implications and Future Directions

The Mesoamerican region is an active and important biogeographical corridor joining the biotas of the Americas (Raven and Axelrod 1975; Méndez-Carvajal and Moreno 2014; Meyer *et al.* 2015). Yet, climate change has rendered coastal habitats in lower Central America, including mangroves and coastal forests, highly vulnerable to extreme meteorological events such as storm surge, ENSO events, sea-level rise and other marine influences (Lopez and Kursar 2007; O. R. Lopez *et al.*, unpubl. data). Central American countries are predicted to experience above 30 % increment in storm surge under future climatic scenarios (Dasgupta *et al.* 2009). This concurs with regional climate change forecasts that consider a great portion of the pacific coastline bordering the Gulf of Panama, to be severely affected by sea-level rise (Mckee and Vervaeke 2018), while for the Caribbean area the impact might be lower. Our research demonstrates that relatively salt-tolerant tree species such as *Pithecellobium*, *Mora*, *Terminalia*, *Thespesia* and *Sterculia* could be readily employed in reforestation strategies along coastal areas.

Seedlings of coastal and inland tropical tree species evaluated in this study showed decreased physiological and growth performance under increased seawater irrigation. However, under salinity, seedlings of species from coastal and dry forest showed superior growth and survivorship in contrast with inland or wet forest species. Despite the limitation of irrigation experiments, as the ‘effective’ salinity of the soil is not always clearly defined, our comparative study provides useful information about salinity tolerance among a significant group of tropical woody species and provides a first step towards formulating mitigation strategies (Fig. 7) in view of the potential consequences of sea-level rise in the face climate change in tropical America.

Further studies are critical in addressing the effects of salinity among tropical tree species in relation to their association with certain functional groups such as N_2_-fixing legumes. This highlights the importance to understand salinity tolerance at a broader ecological scale in order to predict plant community shifts along exposed coastal areas. Additionally, underpinning the molecular basis for the expression of key salinity tolerance genes among tropical woody species is of critical importance.

## Supporting Information

The following additional information is available in the online version of this article—

**Figure S1.** Stem height reduction at 20 (A), 40 (B) and 60 % (C) of seawater irrigation treatment (±SE) in coastal and inland species.

**Figure S2.** Absolute relative growth rates (±SE) for all studied species across seawater treatments. Panels are arranged in relation to Table 1.

**Figure S3.** Reduction in relative growth rate (RGR) given as percentage of control seedlings under 90 % of seawater treatment (±SE) for coastal and inland species.

**Figure S4.** Cladogram representing species salinity tolerance ranking according to a hierarchical clustering analysis using all response parameters, including *g*_s_ and *A*_max_, under 90 % seawater treatment. Within each clade, species are arranged by ascending ranking of salinity tolerance. Note: hierarchical clustering analysis excludes species with missing values.

**Appendix S1.** *A*_max_ and *G*_s_.

**Appendix S2.** Leaf-height-mortality.

plz062_suppl_Supplementary_Information

plz062_suppl_Supplementary_Response_To_Reviewer

plz062_suppl_Supplementary_Tabla_Madre_Amax_Y_Gs

plz062_suppl_Supplementary_Tabla_Madre_Leaf-Height-Mortality

## Sources of Funding

This research was financially possible through a SENACYT-IFARHU doctoral scholarship to A.D.S., and a grant from the Dirección de Investigación y Desarrollo (EFA11-015) from the Secretaría Nacional de Ciencia Tecnología e Innovación (SENACYT) and the support of the Sistema Nacional de Investigación (SNI) awarded to O.R.L.

## Contributions by the Authors

O.R.L. and K.W. conceived and designed the study, facilitated the logistics, contributed to the analysis and final writing. A.D.S. conducted the experiments, analyzed the data and led the early writing. Y.G. contributed to the fieldwork.

## Conflict of Interest

None declared.
