# Supplementary material for: Seedling responses to salinity of 26 Neotropical tree species
Source: AoB Plants. 2019 Nov 25;11(6):plz062. doi: 10.1093/aobpla/plz062 (PMC6876892; doi:10.1093/aobpla/plz062)

Supporting Information

**Figure S1**.

Stem height reduction at 20 (A), 40 (B) and 60% (C) of seawater irrigation treatment (± s.e.) in coastal and inland species.


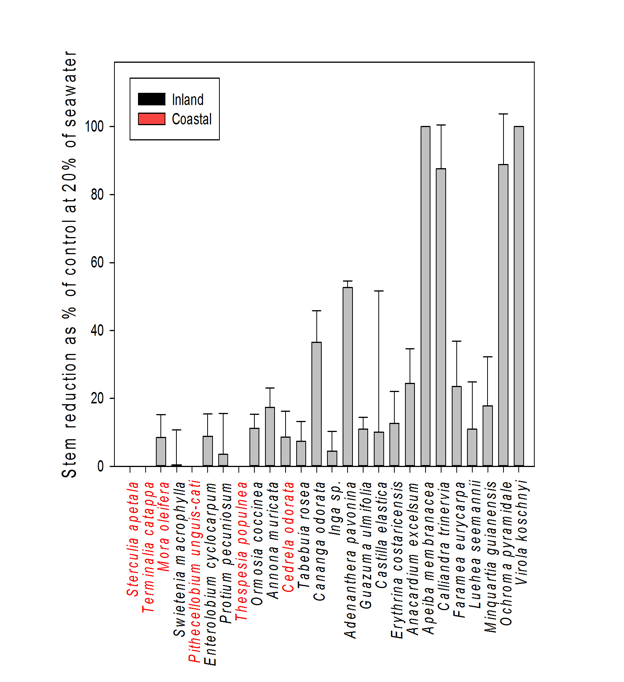


A


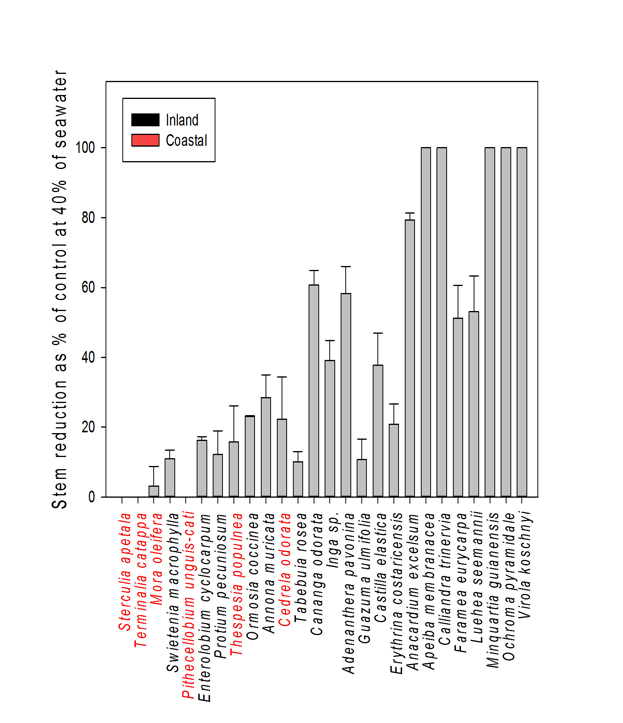


B


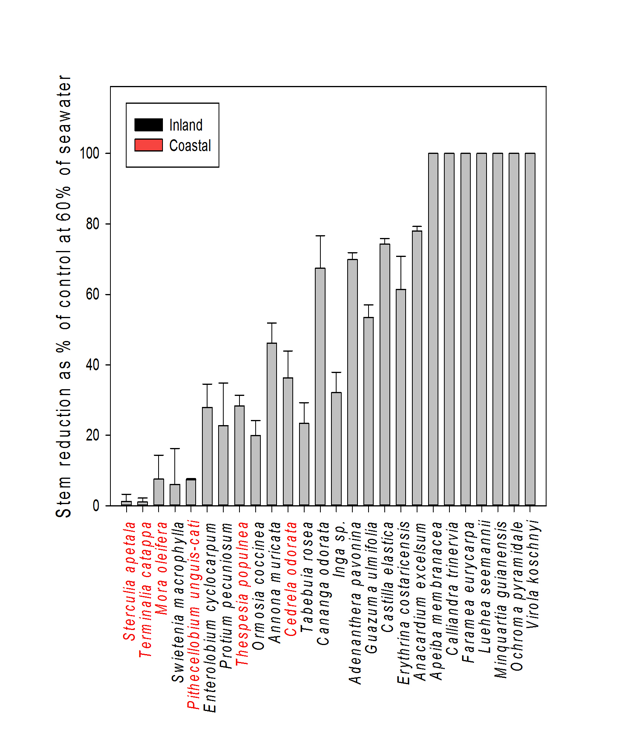


C

**Figure S2**.

Absolute relative growth rates (± s.e.) for all studied species across seawater treatments. Panels are arranged in relation to Table 1.


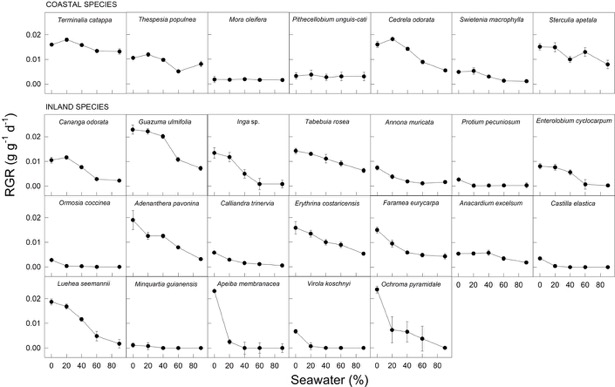


**Figure S3**.

Reduction in RGR given as percentage of control seedlings under 90% of seawater treatment (± s.e.) for coastal and inland species.


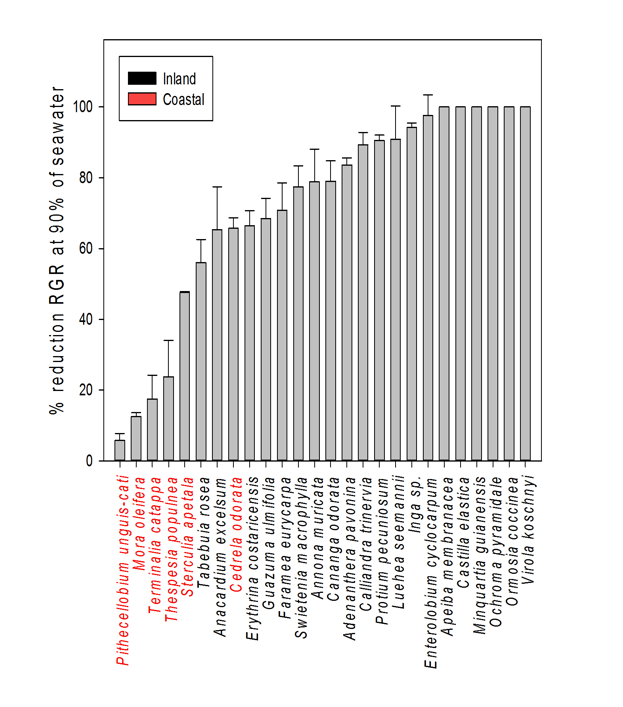


**Figure S4**.

Cladogram representing species salinity tolerance ranking according to a hierarchical clustering analysis using all response parameters, including *g*_s_ and *A*_max_, under 90% seawater treatment.  Within each clade, species are arranged by ascending ranking of salinity tolerance. Note: hierarchical clustering analysis excludes species with missing values.


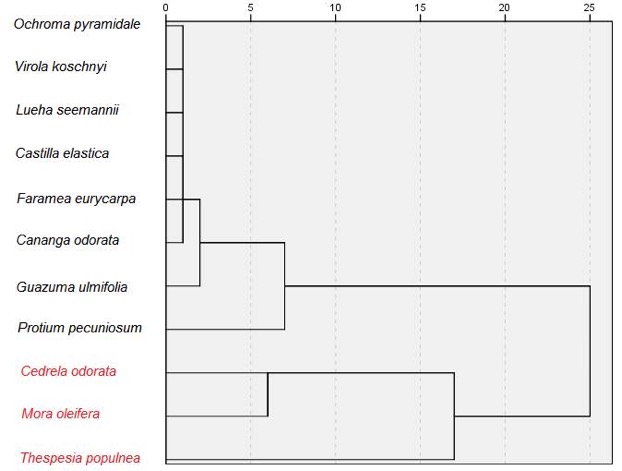

Supplement: plz062_suppl_Supplementary_Information [file plz062_suppl_supplementary_information.docx]
