# Supplementary material for: Seedling responses to salinity of 26 Neotropical tree species
Source: AoB Plants. 2019 Nov 25;11(6):plz062. doi: 10.1093/aobpla/plz062 (PMC6876892; doi:10.1093/aobpla/plz062)
Supplement: plz062_suppl_Supplementary_Response_To_Reviewer [file plz062_suppl_supplementary_response_to_reviewer.docx]

Recommendation: Accept Revision Required 


Referee #1 Evaluations: 
Recommendation: Major changes needed 

Referee #1 (Comments for the Author (Required)): 

Review of 18141S1 for AoB Plants 

This manuscript seeks to determine growth responses to salinity treatments across 26 species from both inland and coastal habitats. They also aggregate the data to assess a salinity ranking for the species in the study. 

I reviewed the previous version of this manuscript and think the manuscript is significantly improved as a result of that round of comments. The presentation and writing is now much clearer. The introduction is now much clearer and sets up the case for the manuscript. The clearly stated objectives and hypothesis allow for the reader to understand the goal of the manuscript. There is also added information about species selection and using this for interpretation in the discussion. 

I still have a few key questions and comments outlined below. My biggest concerns center around the justification for how the ranking analysis was done and the presentation of the data. 

Specific comments 
1. Line 51: No semicolon needed.

R. Done

2. Line 95: word missing 
R. Corrected. We changed these sentences around to read: “Plants were treated with 150 ml of 20, 40, 60 and 90% of seawater, while control plants (0% seawater) were irrigated with 150 ml of tap water, respectively, once a week.

3. Lines 116-117: How did you decide when the "end of experiment" was? 
R. Corrected. In line 100 (former version) we stated that the experiments lasted 60 days. We have moved this sentence to line 116-117 in order to better contextualize the survivorship estimation at the end of the experiment. We did not “decided” when to end the experiments, all species were uniformly brought up to nearly 60 days of treatment (+ or – 2 days). This has been added to the current version.

4. Results: All p values should be accompanied by their test statistic with degrees of freedom (F). Also it is unnecessary to write "ANOVA" each time in parentheses.

R. We revised all the statistics and adjusted some of the analysis (changes in red in the main text).

5. Lines 156-164: The manuscript describes results from the 20% seawater treatment. Figure 1 just shows the results of the 90% seawater treatment. I appreciate that the authors are trying to save space, but I would suggest including figures identical to figure 1 using the other treatments (0, 20, 40, 60 % seawater) and include this in the supplemental information.

R. Done. We have added the remaining figures for the 20, 40 and 60% seawater treatments to the Supplemental Data section and made reference to the availability of those figures in the main text. While we described some results from lower seawater concentrations throughout the manuscript, the 90% treatment results provides a clearer picture of the difference between habitats.

6. Figure 1, 2, 4, and 5. Shouldn't all of these have error bars?

R. Done. We have redrawn these figures to express their standard errors.

7. Figure 4 feels somewhat repetitive from Figure 3 and could probably be moved to the supplemental.

R. Done. We have moved Figure 3 from the main text to the Supplemental Data section and adjusted the numeration accordingly.

8. Figure 5.This is interesting. You ultimately end up with a group of species that die no matter what in this treatment. You then have the opposite, a group of species that survive (mostly coastal species). Then you have a group in the middle where some live and some die. Exploring the mechanism for "why these species" a little more in the discussion would improve the manuscript. Does this pattern hold with the ranking analysis?

R. We have arrived at a similar observation while conducting the hierarchical analysis (now in Figure 7) in early versions of the manuscript. This is, there appears to be three clusters; “tolerant”, “intolerant” and in the middle the “sensitive” species. However, we considered that differentiating them beyond a “tolerant” and “sensitive” dichotomy will add bias to our interpretation as all these species are known as glycophytes. Additionally, introducing a third category might be a bit distracting from the main objective which is to compare salinity tolerance in coastal vs inland species. However, following the reviewer suggestion, we have added to the discussion the potential link between drought tolerance and salinity (lines 287-293). Also, in lines 295 to 306 we already explore some plausible mechanisms for some other species, for which we have information about traits and their natural history.  

9. Figure 9. I do not entirely understand how this figure was derived or why only 3 species are on it? Overall, I am not sure it adds value.

R. We agree, this figure might not add value to the manuscript. We adapted a “crop-yield” salinity threshold diagram from Mass and Hoffman 1977 to better complement the information presented in the HA. The figure depicts the expected threshold tolerance based on three levels “intolerant”, “sensitive” and “tolerant” where any tropical tree species, exposed within the salinity range use in our study, could be “mapped” as a relevant number of species (26) were used to delineate such thresholds. We have removed the figure and any reference to it from the manuscript.

10. Lines 198-199: This sentence is confusing. You can remove "by and large". More importantly, it looks like for some of the species that inland species had greater reduction in response to high salinity. The related figures (6 & 7) tell a much more complicated story than this statement.

R. Agree. We removed “by and large” (now line 200).

11. Lines 214-218. So this analysis used growth rate, leaf number, and mortality but not gs and Amax? In addition, the analysis only uses the 90% treatment? This feels a little bit like the authors selected the parameters and treatments that produced a result that made sense. At minimum, the manuscript needs to thoroughly justify the rationale for why these decisions were made.

R. We disagree. First, we did not arbitrarily selected the parameters used in the hierarchical analysis (HA). The decision to exclude the gas exchange parameters rest on the fact that this will contribute to “missing data” as a significant number of species where not measured. Most available HA algorithms are particularly sensitive to missing data, therefore we are only using the growth response parameters (219-221). The objective was to use this type of clustering analysis as an exploratory tool with the objective to visually represent our results in a concise way. Previously, we have ranked the species based on a compound index using all variables, but this approach was criticized for being arbitrary. We recognize, as it is common with exploratory toll such this, that the analysis involves arbitrary decisions and the choosing of the 90% treatment is based on the fact that this represents the clustering that best mapped our findings. We have added some justification in the methods as well as in the discussion of our results.

12. Lines 256-260. These sentences make a statement that could (and perhaps should) be tested. Is there a relationship between changes to RGR and changes to Amax or gs? If there is a relationship between these two then this should be added as a figure. If there is not, then this statement should be removed.

R. We tested the relationship between changes in RGR in relation to Amax across the measured species by treatments. While the relationship is positive in all cases, only Amax at 30 days explained 30% of the variance in RGR under the 20% seawater treatment (*r*^2^ = 0.29 p = 0.0498). Although the evidence is weak, we have modified the text in the discussion (lines 257-260) to acknowledge exploration of this relationship as it is well recognized in the stress physiology literature. The lack of relationship among these variables may respond to the fact that RGR is an integrated variable and Amax represents an instantaneous measure of the photosynthetic performance after 1 and 2 months. The usefulness of the *A*_max_ data relies on the behavior of photosynthesis across the experiment (at 0, 30 and 60 days) in relation to the treatments. This is addressed in the Results section (*Stomatal conductance and maximum photosynthetic rate*) and the following lines in the same paragraphs in the discussion.
